# Functional relationships between plasmids and their significance for metabolism and symbiotic performance of *Rhizobium leguminosarum* bv. *trifolii*

**DOI:** 10.1007/s13353-014-0220-2

**Published:** 2014-05-17

**Authors:** Grażyna Stasiak, Andrzej Mazur, Jerzy Wielbo, Małgorzata Marczak, Kamil Żebracki, Piotr Koper, Anna Skorupska

**Affiliations:** Department of Genetics and Microbiology, Maria-Curie Skłodowska University, 19 Akademicka St., 20-033 Lublin, Poland

**Keywords:** *Rhizobium*, Megaplasmids, Symbiosis, Metabolism, Plasmid curing

## Abstract

*Rhizobium leguminosarum* bv. *trifolii* TA1 (RtTA1) is a soil bacterium establishing a highly specific symbiotic relationship with clover, which is based on the exchange of molecular signals between the host plant and the microsymbiont. The RtTA1 genome is large and multipartite, composed of a chromosome and four plasmids, which comprise approximately 65 % and 35 % of the total genome, respectively. Extrachromosomal replicons were previously shown to confer significant metabolic versatility to bacteria, which is important for their adaptation in the soil and nodulation competitiveness. To investigate the contribution of individual RtTA1 plasmids to the overall cell phenotype, metabolic properties and symbiotic performance, a transposon-based elimination strategy was employed. RtTA1 derivatives cured of pRleTA1b or pRleTA1d and deleted in pRleTA1a were obtained. In contrast to the in silico predictions of pRleTA1b and pRleTA1d, which were described as chromid-like replicons, both appeared to be completely curable. On the other hand, for pRleTA1a (symbiotic plasmid) and pRleTA1c, which were proposed to be unessential for RtTA1 viability, it was not possible to eliminate them at all (pRleTA1c) or entirely (pRleTA1a). Analyses of the phenotypic traits of the RtTA1 derivatives obtained revealed the functional significance of individual plasmids and their indispensability for growth, certain metabolic pathways, production of surface polysaccharides, autoaggregation, biofilm formation, motility and symbiotic performance. Moreover, the results allow us to suggest broad functional cooperation among the plasmids in shaping the phenotypic properties and symbiotic capabilities of rhizobia.

**Electronic supplementary material:**

The online version of this article (doi:10.1007/s13353-014-0220-2) contains supplementary material, which is available to authorized users.

## Introduction

*Rhizobium leguminosarum* bv. *trifolii* (*Rlt*) is a soil bacterium establishing a symbiotic relationship with clover, *Trifolium* spp. and inducing the formation of root nodules on the host plant, in which bacteria differentiate into nitrogen-fixing bacteroids. Inside the nodules, atmospheric nitrogen is converted into ammonia and used by the plant as a nitrogen source. The interaction between rhizobia and leguminous plants shows a high degree of specificity, and successful infection of the roots is based on the exchange of molecular signals between the host plant and the microsymbiont (Dénarié et al. 1996; Long 1996; Jones et al. 2007). The specificity of the interaction between symbiotic partners is mediated largely by the Nod factor (NF), a substituted lipochitooligosaccharide molecule synthesised by the action of rhizobial nodulation (*nod*) genes, usually located on one of the plasmids (then called a symbiotic plasmid, pSym) or on the chromosome as symbiotic islands (Long 1996; Masson-Boivin et al. 2009). The NF elicits a series of physiological and developmental responses in the host plants, leading to nodule organogenesis (Perret et al. 2000). Chemical modifications of the NF further determine the host range (Dénarié et al. 1996). Besides the pSym, other parts of the rhizobial genome, especially non-symbiotic plasmids, may influence the symbiotic performance of bacteria by encoding additional factors such as proteins and cell surface polysaccharides that affect competitiveness and nitrogen fixation (Skorupska et al. 2006; Wielbo et al. 2007, 2010; López-Guerrero et al. 2012; Sorroche et al. 2012).

Challenging environments, such as soil, in which resources are scarce and living conditions fluctuant, are frequently dominated by bacterial species with large and complex genomes. Rhizobial genomes, especially in the *R. leguminosarum* species, are large and multipartite, composed of a chromosome (core genome) and plasmids (accessory genome); the latter may comprise up to 35–45 % of the total genome (González et al. 2006; Young et al. 2006; Mazur et al. 2011b). In contrast to chromosomes, plasmids are considered as usually poorly conserved, flexible and heterogeneous in size and gene content. Variations in the location of chromosomal and extrachromosomal genes can be observed even in small rhizobial populations (Crossman et al. 2008; Mazur et al. 2011b; López-Guerrero et al. 2012). Some of the rhizobial extrachromosomal replicons referred to as chromids are characterised by a plasmid-type replication system but differ from plasmids in their GC content and codon usage, which are similar to those of the chromosome. Chromids were also distinguished on the basis of the presence of some genetic elements essential for growth under all conditions, which makes them impossible to be eliminated from the parental strains (Harrison et al. 2010; Landeta et al. 2011). On the other hand, plasmids are considered dispensable for growth and survival in natural habitats; these plasmids can be eliminated from the cells and are recognised as “recently” recruited, e.g. by horizontal DNA transfer (Petersen et al. 2013). Recently, the elimination of replicons designated as chromids on the basis of in silico analyses has been reported (Cheng et al. 2007). Petersen et al. (2013) modified the definition of chromids and proposed that replicons prone to elimination but indispensable for the survival in natural habitats should be regarded as essential “sensu lato”, whereas those which cannot be eliminated because of the gene content belonging to the minimal essential gene set (Glass et al. 2006) should be regarded as chromids “sensu stricto”.

It was shown that extrachromosomal replicons conferred significant metabolic versatility to rhizobia, which is important for their adaptation in the soil and nodulation competitiveness (Wielbo et al. 2007, 2010; Mazur et al. 2013). Up to now, several factors that contribute to rhizosphere colonisation and nodulation, such as motility and chemotaxis (Yost et al. 1998), metabolism of carbon and energy sources (Baldani et al. 1992; Oresnik et al. 1998; Yost et al. 2004, 2006; Guerreiro et al. 1998; Ormeño-Orrillo et al. 2008; Ramachandran et al. 2011; Ding et al. 2012), bacteriocin production (Hirsch et al. 1980; Oresnik et al. 1999; Venter et al. 2001), synthesis of vitamins (Miranda-Ríos et al. 1997; Villaseñor et al. 2011) and production of cell surface polysaccharides (Brom et al. 1992, 2000; García-de los Santos and Brom 1997; Skorupska et al. 2006), have been identified to be localised in plasmids in *Rhizobium* spp. (López-Guerrero et al. 2012; Mazur and Koper 2012, for a review). Besides the contribution of individual plasmids to the adaptive potential of rhizobia, their interactions also seem to be important. For example, the symbiotic capability of *R. etli* CFN42 was shown to be determined by genes located on pSym, but genes such as *lps* or *fix* also required for effective symbiosis are located on other plasmids, and for the mobilisation of plasmid pRet42d (pSym), a conjugative plasmid pRet42a is essential (Brom et al. 2000). Despite extensive research in the field, data concerning how the accessory genome itself and functional relationships between extrachromosomal replicons shape the phenotype and metabolism of the bacterial cell are still scarce.

The genome of *R. leguminosarum* bv. *trifolii* TA1 (RtTA1) is composed of five replicons: a chromosome and four megaplasmids (pRleTA1a–pRleTA1d), ranging in size from 476 to 808 kb (Król et al. 2008; Mazur et al. 2011a). The plasmids have been characterised as RepABC-type replicons, and two of them, i.e. pRleTA1b and pRleTA1d, were predicted to be chromid-like replicons on the basis of their GC content and codon usage (Mazur et al. 2011a, b). The smallest plasmid, containing genes determining the ability to establish a nitrogen-fixing symbiosis, was identified as pSym (Król et al. 2008). Besides the *repA*, *repB* and *repC* replication/partition genes, only a small number of markers of individual RtTA1 plasmids was identified and sequenced (Król et al. 2008; Mazur et al. 2011a, b).

The aim of this work was to investigate the contribution of individual RtTA1 plasmids to the overall phenotype, metabolism and symbiotic performance, employing the elimination approach. A transposon-based strategy combined with a direct selection for plasmid loss was employed (Hynes et al. 1989; Brom et al. 1992). RtTA1 derivatives cured of or deleted in three individual plasmids have been obtained. Despite many attempts, curing of the entire pRleTA1c and pRleTA1a plasmids was unsuccessful. Important phenotypic traits of the plasmid-cured or -deleted strains, i.e. growth kinetics, metabolic capabilities, autoaggregation, motility, biofilm formation, production of surface polysaccharides and symbiotic performance, were established and revealed some functional relationships between the RtTA1 plasmids.

## Materials and methods

### Bacterial strains and growth conditions

The bacterial strains and plasmids used in this work are listed in Table 1. *Rhizobium* strains were grown in 79CA (Vincent 1970), tryptone yeast (TY) or M1 media (Sambrook et al. 1989) at 28 °C for 2–3 days. *Escherichia coli* strains were grown in Luria-Bertani (LB) medium at 37 °C. Antibiotics were used at the following concentrations (μg ml^−1^): ampicillin (Ap) 100–200, chloramphenicol (Cm) 30, gentamicin (Gm) 15, kanamycin (Km) 40, rifampicin (Rf) 40, streptomycin (Sm) 200–300 and tetracycline (Tc) 10. To select for plasmid loss, the media were supplemented with 5 % sucrose.

**Table 1:** Bacterial strains and plasmids used in this study

Strain or plasmid	Relevant characteristic	Source or reference
*R. leguminosarum* bv. *trifolii*	TA1, wild type, Sm^r^, Rif^r^	Chakravorty et al. (1982)
*R. leguminosarum* bv. *viciae*	3841, Sm^r^	Young et al. (2006)
RtTA101, RtTA105	TA1 cured of pRleTA1d (pd^−^)	This work
RtTA102	TA1 cured of pRleTA1b (pb^−^)	This work
RtTA106	TA1 deleted in pRleTA1a (pa∆), the size of residual pRleTA1a was estimated at about 300 kb	This work
RtTA107	TA1 deleted in pRleTA1a (pa∆), the size of residual pRleTA1a was estimated at about 400 kb	This work
RtTA109	TA1 deleted in pRleTA1a (pa∆), the size of residual pRleTA1a was estimated at about 350 kb	This work
*Escherichia coli*	S17-1, 294 RP4-2-Tc::Mu::Tn7 integrated into chromosome	Simon et al. (1983)
pMH1701	Tn5-B12S (*sacB*, Mob^+^), Amp^r^, Tc^r^, Nm^r^	Hynes et al. (1989)
pJQ118	Tn5-M8S (*sacB*, Mob^+^), Amp^r^, Tc^r^, Gm^r^	Quandt et al. (2004)

### Bacterial mating and screening for plasmid loss

Bacterial mating experiments were performed as described by Simon et al. (1983). Briefly, an *E. coli* S17-1 donor strain harbouring pMH1701 or pJQ118 plasmids (Hynes et al. 1989; Quandt et al. 2004) was grown to the early exponential phase in LB liquid medium with appropriate antibiotics, washed twice with sterile water and resuspended in water. The RtTA1 recipient strain was grown in 79CA to the late exponential phase and mixed with the donor strain at a ratio of 1:2. The mating mixture (100 μl) was loaded onto a Millipore filter (0.45 μm pore size), placed on 79CA agar and incubated overnight at 28 °C. The bacteria were then rinsed out from the filter with 79CA and selected on 79CA agar plates with kanamycin (pMH1701) or gentamicin (pJQ118) and rifampicin (for the donor counter selection). Cultures derived from single colonies of transconjugants were grown in liquid 79CA for 14–17 h at 28 °C and plated in dilutions on TY with 5 % sucrose. Colonies appearing on TY with sucrose were screened for the content of plasmids using the method of Eckhardt (1978).

### DNA methods

Standard techniques were used for DNA labelling, Southern hybridisation and agarose gel electrophoresis (Sambrook et al. 1989). DNA probes for Southern hybridisation were obtained by polymerase chain reaction (PCR) amplification with RtTA1 genomic DNA as a template and appropriate primers. Primers and probes used in the study were described previously by Mazur et al. (2011b). Hybridisation probes were labelled with the non-radioactive DIG DNA Labeling and Detection Kit (Roche). Hybridisations were performed at high stringency at 42 °C using 50 % formamide in pre-hybridisation solutions.

### Plasmid analyses

For the analyses of the plasmid content of RtTA1 derivatives, the Eckhardt (1978) and pulsed-field gel electrophoresis (PFGE) techniques were used. PFGE was performed using the contour-clamped homogenous electric field mode with the Bio-Rad system (model CHEF-DR III). DNA samples were separated in 0.9 % agarose gels in 0.5 × TBE buffer at 14 °C, with switch times of 30–85 s, angle 120° and voltage gradient 6 V/cm for 22 h. The sizes of deleted derivatives of the RtTA1 plasmids were estimated using *R. leguminosarum* bv. *viciae* strain 3841 (Young et al. 2006) as a standard for replicon size.

### Growth kinetics testing

Plasmid-cured or -deleted derivatives of RtTA1 were grown with shaking (150 rpm) in 79CA medium for 48 h. Afterwards, the cells were washed in sterile water, suspended to OD_550_ 0.01 in TY, 79CA and M1 medium [the latter was supplemented with a vitamin mixture according to Brown and Dilworth (1975)], and various carbon sources to 1 %. The OD_550_ of the cultures grown with shaking at 28 °C was monitored at regular time intervals (24 h) up to 72 h. The measurements were done in triplicate.

### Analysis of the utilisation of carbon and energy sources

The Biolog GN2 MicroPlate (Biolog, Hayward, CA, USA) test (Bochner 2009) was conducted according to the manufacturer’s instructions. Briefly, rhizobia grown overnight at 28 °C on TY agar medium were collected and washed with sterile water. Next, the pellet was diluted in water to an initial OD_550_ of 0.1 (approximately 10^8^ cells ml^−1^) and 150 μl of the rhizobial suspension was inoculated into each well of the GN2 microplate. The plates were incubated for 72 h at 28 °C and colour development (absorbance at 590 and 750 nm) in the wells was recorded using a Benchmark Plus microplate reader (Bio-Rad Laboratories, USA). The conversion of colourless tetrazolium violet to a purple-coloured compound meant a normal process of respiration (positive phenotype), whereas, when the phenotype was negative, the wells remained colourless. The optical density values of the Biolog microplate wells were corrected using background colour developed in the control well.

### EPS isolation

Bacteria were grown in 79CA with 1 % glycerol at 28 °C for 48 h. EPS was precipitated from 1-ml culture supernatants with three volumes of 96 % ethanol. The precipitate was collected by centrifugation, dried, resolved in water and submitted for the total carbohydrate analysis according to Loewus (1952). The total sugar content was calculated as glucose equivalents and was the result of four independent replicas.

### Analysis of autoaggregation

Autoaggregation of bacteria was measured as described by Sorroche et al. (2012). Bacteria were grown at 28 °C for 24 h, diluted 100-fold in TY medium and incubated for 48 h. Afterwards, 5 ml of bacterial cultures were transferred to new tubes, measured for the absorbance at 600 nm (A_0_) and then incubated at 4 °C for 24 h without agitation. Next, 0.2 ml of the upper suspension was transferred to microplate wells and OD_600_ (A_t_) was measured in eight independent experiments. The autoaggregation percentage was expressed as follows: 1 – (A_t/_A_0_) × 100 (Sorroche et al. 2012).

### Motility and biofilm formation assay

The motility assay was conducted in 0.3 % M1 agar medium with glycerol. A 5-μl culture grown in M1 medium at 28 °C for 24 h to an OD_600_ of 0.4 was stabbed into plates with TY medium. The plates were incubated at 28 °C for 3 days, and bacterial growth from the point of inoculation was measured. The motility assay was done in triplicate. Biofilm formation was assessed as described previously by Fujishige et al. (2006) and Sorroche et al. (2012). Briefly, rhizobial strains were grown in TY medium at 28 °C for 48 h. The cultures were diluted to an OD_600_ of 0.1 in TY, inoculated into 96-well microtitre plates in 100-μl aliquots and incubated with agitation (100 rpm) at 28 °C for 24 h. Next, bacterial growth was assessed by measuring the OD_600_. The planktonic cells were gently removed, stained for 15 min with 180 μl 0.1 % crystal violet and then rinsed three times with water. Biofilm formation was quantified by the addition of 150 μl of 95 % ethanol and measurement of the absorbance at 560 nm was done in a microplate reader MicroElisa Autoreader. The experiment was performed in triplicate.

### Plant tests

Seeds of red clover (*T. pratense* L. cv. Rozeta) were surface-sterilised and germinated on nitrogen-free medium, as described earlier (Vincent 1970). Two-day-old clover seedlings were planted in sterile nitrogen-free slants (one per tube) and allowed to grow for 4 days before being inoculated with 0.2 ml of *Rlt* cell suspension at an approximate density of 1.0 × 10^9^ cells/ml. The plants were grown in a greenhouse under natural light supplemented with an artificial light regime (14/10 h light/dark). After 5 weeks, the plants were harvested and fresh mass of shoots/roots and nodule formation were examined. Twenty clover plants were used for each strain. To assess the stability of the plasmid-cured RtTA1 derivatives, the rhizobia were isolated from a few nodules and their plasmid content was examined.

### Statistical analyses

The results of RtTA1 derivatives: growth kinetics, EPS production, plant tests, as well as autoaggregation, motility and biofilm formation assays, were submitted for statistical analyses, which were performed with STATISTICA software, using one-way analysis of variance (ANOVA) and the Tukey test at a significance level of *p* < 0.05.

## Results

### RtTA1 plasmid elimination

**Fig. 1:**
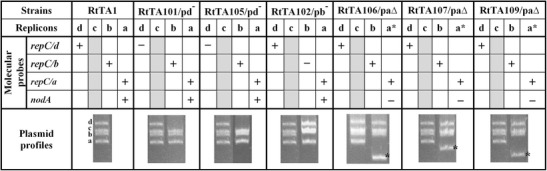
Genetic characteristic of *R. leguminosarum* bv. *trifolii* TA1 and its plasmid-cured or -deleted derivatives. Plasmid profiles of all strains were obtained with agarose gel electrophoresis using the Eckhardt lysis procedure and are shown at the bottom of the table. Particular replicons of RtTA1 and its derivatives are marked with letters a, b, c and d, which refer to pRleTA1a, pRleTA1b, pRleTA1c and pRleTA1d plasmids, respectively. The strains shown are: RtTA1, the wild type, RtTA101 and RtTA105 (pRleTA1d-cured), RtTA102 (pRleTA1b-cured), and RtTA106, RtTA107 and RtTA109 (pRleTA1a-deleted). The *asterisks* show the positions of pRleTA1a deletion derivatives. The replicons of the strains were PFGE separated and probed with *repC* genes specific for individual plasmids and *nodA* specific for the symbiotic plasmid; “+” means positive hybridisation and “−” negative hybridisation. The column with pRleTA1c was shaded grey as no hybridisation with probes specific for this plasmid was performed

The RtTA1 laboratory strain contains four extrachromosomal replicons, designated pRleTA1d, pRleTA1c, pRleTA1b and pRleTA1a (Fig. 1), with approximate molecular sizes of 808, 653, 603 and 476 kb, respectively. To assess the impact of particular plasmids on the RtTA1 phenotype, symbiotic capabilities and metabolism, an elimination experiment was undertaken using the transposon-based (Tn5*sacB*) method combined with a direct selection for plasmid loss (Hynes et al. 1989; Brom et al. 1992; Quandt et al. 2004). It should be noted that the total elimination of individual RtTA1 plasmids using incompatibility mechanisms was not possible (Mazur et al. 2011a). Sucrose-resistant colonies characterised by the loss of resistance to antibiotics carried by the vectors were screened for plasmid elimination by Eckhardt’s method. The RtTA1 derivatives with changes in their plasmid profile, i.e. plasmid loss or partial deletion, were further separated by PFGE and probed with *repC* genes encoding replication/partition proteins specific for individual plasmids and the *nodA* gene, which is specific for the pRleTA1a symbiotic plasmid.

RtTA1 derivatives that lost entire pRleTA1d or pRleTA1b were obtained (Fig. 1, Table 1). In the case of pRleTA1a (pSym), several derivatives with partial plasmid deletions were isolated. Complete elimination of this replicon was not achieved. The pRleTA1d and pRleTA1b elimination was confirmed by negative results of PCR amplification of the plasmid markers (*prc*, *hlyD* for pRleTA1d and *nadA*, *minC* for pRleTA1b) and negative results of hybridisation with respective *repC* probes. The pSym-deleted derivatives were identified on the basis of negative hybridisation with *nodA* and positive with respective *repC* probe (*repC*/a), which hybridised to a replicon smaller than pSym and was observed in each pSym-deleted strain (Fig. 1, Table 1). Despite repeated attempts performed under various experimental conditions (e.g. elevated temperature of growth), the elimination or at least partial deletion of pRleTA1c was unsuccessful. The genetic characteristics of six RtTA1 derivatives, i.e. analysis of their plasmid content and hybridisation results with probes specific for individual replicons, are shown in Fig. 1.

### Symbiotic properties of RtTA1 derivatives cured or deleted in individual plasmids

**Fig. 2:**
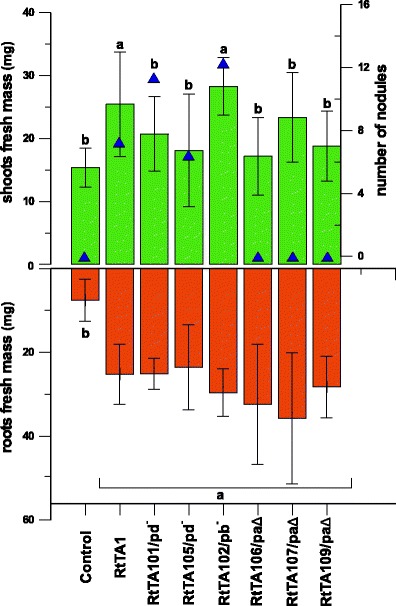
Symbiotic capabilities of RtTA1 derivatives cured or deleted in individual plasmids inoculated on clover plants (*T. pratense*). The *triangles* indicate the number of nodules/plant, the *green bars* indicate shoot masses of plants (mg/plant) and the *orange bars* indicate fresh mass of roots (mg/plant). The *bars* labelled with various letters represent shoot and root mass values which are significantly different at *p* < 0.05 and the *extended bars* represent the standard deviation

pRleTA1a has been previously identified as a symbiotic plasmid containing a *nod*–*fix* region responsible for nodulation and nitrogen fixation (Król et al. 2008; Mazur et al. 2011b). The RtTA106, RtTA107 and RtTA109 strains with deletions in pRleTA1a and displaying negative hybridisation with the *nodA* probe lost the ability to nodulate clover, confirming the indispensability of pRleTA1a for the symbiotic interaction. Fresh masses of plant shoots inoculated with these derivatives were significantly lower (average 20.24 mg/plant, Tukey test *p* < 0.05) than of clover plants infected with the RtTA1 wild type (25.45 mg/plant) (Fig. 2).

The elimination of pRleTA1b did not affect the nodulation ability of the RtTA102 derivative: its symbiotic performance (nodule number 12.3/plant, average fresh shoot mass 28.3 mg/plant) was even higher than that of the RtTA1 wild type (nodule number 7.3/plant).

The plants inoculated with RtTA101 and RtTA105 cured of pRleTA1d showed significantly lower fresh masses of shoots (average 20.5 mg/plant, in comparison to 25.45 mg/plant for wild type) and a nodule number similar to that of the wild type (average 8 nodules/plant). However, in comparison to RtTA1, their nodulation kinetics was different, as the nodules appeared on clover roots with delay (not shown). Furthermore, the weight as well as the yellowish colour of clover shoots inoculated with the pRleTA1d-eliminated strains resembled the ones inoculated with the pSym-deleted derivatives, allowing a conclusion that the symbiotic phenotype of these strains was Nod^+^Fix^−^.

These results not only indicated the indispensability of the pRleTA1a plasmid for effective symbiosis but also pointed out the functional relationship between the pRleTA1a and pRleTA1d replicons in the symbiotic interaction with clover.

### Growth kinetics of RtTA1 plasmid-cured or -deleted derivatives

**Fig. 3:**
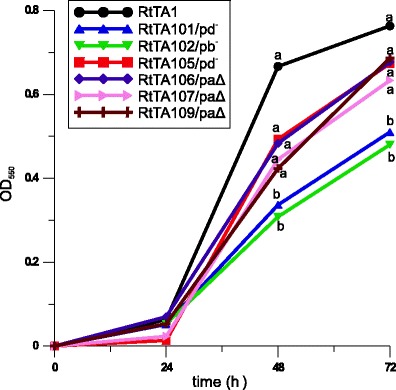
The growth rate of RtTA1 and its plasmid-cured or -deleted derivatives in 79CA medium with 1 % mannitol. The OD_550_ of the cultures grown with shaking was monitored at regular time intervals up to 72 h. The OD_550_ values of the cultures measured after 48 and 72 h labelled with the various letters are significantly different at *p* < 0.05. Standard deviation bars were omitted in order to facilitate chart analysis

All RtTA1 derivatives cured or deleted in individual plasmids grew weaker in comparison to RtTA1 in complete media. In 79CA, the growth of the pRleTA1d-cured (RtTA101 and RtTA105) and pRleTA1b-cured (RtTA102) strains was apparently slower in relation to the wild type strain (Fig. 3). The RtTA106, RtTA107 and RtTA109 derivatives deleted in pRleTA1a grew faster than those cured of pRleTA1d or pRleTA1b and only slightly slower than RtTA1 (Fig. 3); however, only the growth of RtTA101 and RtTA102 differed significantly from the other strains, including the wild type (Fig. 3). The differences in the growth of the strains in TY medium were less apparent, but, still, RtTA102 grew substantially more weakly than the other strains (data not shown). All RtTA1 derivatives were able to grow in M1-NH_4_ minimal medium supplemented with vitamins and mannose as a sole carbon source. In comparison to the other strains, RtTA102 displayed weaker growth in M1-NH_4_ with glycerol and it did not grow in M1 with rhamnose as a sole carbon source (data not shown).

Taken together, it may be concluded that pRleTA1d and pRleTA1b plasmids, but not pSym, substantially affect the RtTA1 growth, which emphasises their importance for cell metabolism.

### Determination of EPS production, autoaggregation and biofilm formation by the RtTA1 derivatives

To further characterise the impact of RtTA1 plasmids on the cell phenotype, we focused on the bacterial extracellular components, which contribute significantly to the rhizobial capabilities important for saprophytic and symbiotic performance. It has been shown that the quality and quantity of surface polysaccharides, particularly exopolysaccharides (EPSs) and lipopolysaccharides (LPSs), produced by rhizobia may influence both their autoaggregation and biofilm formation (Rinaudi and González 2009; Sorroche et al. 2010, 2012). Rhizobial cell-to-surface interaction leading to the formation of biofilm plays a crucial role in root hair infection during symbiosis (Bogino et al. 2013).

**Fig. 4:**
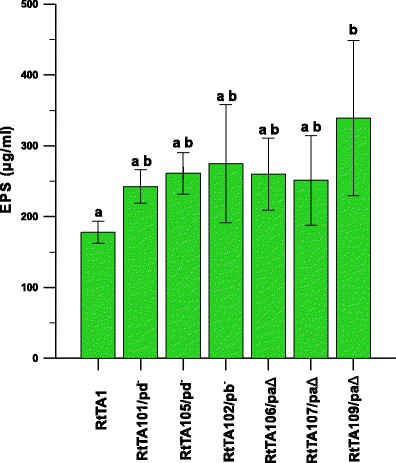
EPS production by RtTA1 plasmid-cured or -deleted derivatives as measured after cultivation in 79CA medium with 1 % glycerol. The total sugars content was calculated in glucose equivalents (μg/ml). The *green bars* labelled with the various letters represent EPS values which are significantly different at *p* < 0.05 and the *extended bars* represent the standard deviation

In *R. leguminosarum* bv. *trifolii* RtTA1, the main gene cluster required for EPS synthesis, i.e. Pss-I, is localised on the chromosome. Other regions plausibly involved in EPS and LPS production were also identified in the RtTA1 genome, with one of them, namely Pss-III, localised in the pRleTA1b plasmid (Król et al. 2007). The exopolysaccharide production by RtTA1 derivatives cured of or deleted in individual plasmids was measured. All RtTA1 derivatives produced slightly higher amounts of EPS in comparison to the RtTA1 wild type (Fig. 4); however, only the amount of EPS observed in the RtTA109 strain deleted in pRleTA1a differed significantly (Fig. 4). Together, the results suggested the existence of a complex regulatory network of EPS synthesis consisting of genes located on different plasmids and especially on pSym.

**Fig. 5:**
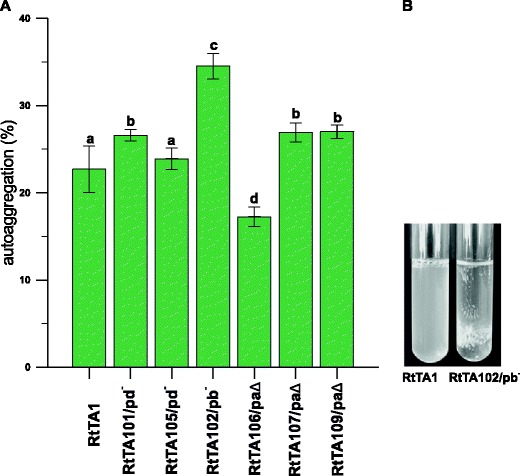
Autoaggregation of RtTA1 plasmid-cured or -deleted derivatives. **a** Quantitative estimation of autoaggregation of strains cultured in 79CA; the autoaggregation is expressed in % according to the following equation: 1 – (A_t_/A_0_) × 100, where A_0_ stands for OD_600_ measured before the non-agitation period and A_t_ is the OD_600_ value after 24 h without agitation. The *green bars* labelled with the various letters represent autoaggregation values which are significantly different at *p* < 0.05 and the *extended bars* represent the standard deviation. **b** Example of growth of the wild type RtTA1 and RtTA102 strains cured of pRlTA1b in 79CA medium

Symbiotic bacteria may develop on plant roots as microcolonies, bacterial aggregates or biofilms (Morris and Monier 2003). Therefore, we analysed the autoaggregation and biofilm formation capabilities of RtTA1 derivatives. Bacterial autoaggregation is a process whereby bacteria physically interact with each other and settle to the bottom in a static liquid suspension (Sorroche et al. 2010, 2012). The level of autoaggregation of RtTA1 derivatives resulting in their sedimentation on the bottom of tubes during growth in 79CA static liquid cultures was measured. Almost all derivatives differed significantly from the wild type; however, only the RtTA102 (pRleTA1b-cured) strain showed substantially increased autoaggregation when compared to RtTA1 (34.5 % RtTA102 vs. 23.6 % RtTA1) (Fig. 5). With the exception of RtTA102, no obvious relation between individual plasmid elimination and cell self-aggregation was noticed.

**Fig. 6:**
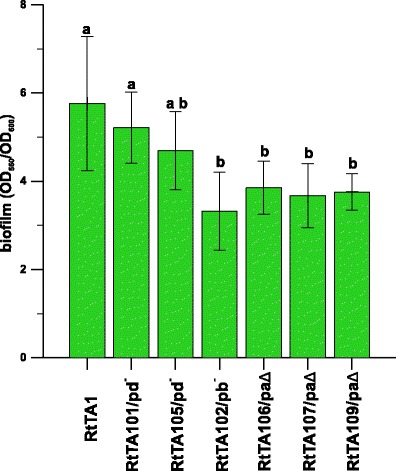
Biofilm formation by the RtTA1 wild type strain and plasmid-cured or -deleted derivatives as assessed by microtitre plate assay. Biofilm formation capabilities were examined after 24 h from inoculation by staining with crystal violet and expressed as the ratio of the amount of crystal violet solubilised by ethanol (OD_560_) to the bacterial growth (OD_550_). The experiment was performed in triplicate and averaged. The *green bars* labelled with the various letters represent biofilm amounts which are significantly different at *p* < 0.05 and the *extended bars* represent the standard deviation

In *Sinorhizobium meliloti*, a positive correlation between autoaggregation and biofilm formation was found, indicating that both processes depend on the same physical adhesive forces (Sorroche et al. 2012). The biofilm formation by the RtTA1 plasmid-cured or -deleted derivatives was examined by the classical crystal violet (CV) method employing microtitre plastic plates (Fujishige et al. 2006) (Fig. 6). In contrast to self-aggregation, the highest biofilm formation was noticed for the RtTA1 wild type (OD_560_/OD_600_ ratio 5.8), as well as for the RtTA101 and RtTA105 strains cured of pRleTA1d (OD_560_/OD_600_ ratios 5.2 and 4.7, respectively). The pRleTA1a deletion in strains RtTA106, RtTA107 and RtTA109 resulted in a significant (*p* < 0.05) reduction of the biofilm amount (OD_560_/OD_600_ ratios 3.85, 3.67 and 3.8, respectively). A substantially diminished amount of biofilm (statistically important difference, *p* < 0.05) was also observed for RtTA102 (OD_560_/OD_600_ ratio 3.3), but no correlation between self-aggregation of the cells and biofilm formation was determined. It may be concluded that the presence of both pRleTA1a and pRleTA1b is important for biofilm formation, while pRleTA1d plays an insignificant role in this process.

**Fig. 7:**
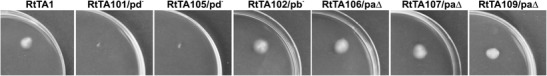
Swimming motility of RtTA1 wild type strain and plasmid-cured or -deleted derivatives. Bacterial growth from the point of inoculation in M1 agar medium (0.3 %) was measured after 3 days

Bacterial motility is one of the critical factors for the establishment of symbiosis under natural soil conditions (Morris and González 2009; Tambalo et al. 2010). In *S. meliloti*, the regulatory proteins MucR, ExoR and ExpR, which play essential roles in EPS synthesis, also seem to be engaged in motility, indicating that the regulation of these two processes may be coupled (Hoang et al. 2008). From among the derivatives studied, the pRleTA1d-cured strains were negative in the motility test (Fig. 7). The swimming zones of RtTA101 and RtTA105 after growth on 79CA semisolid medium for 72 h were significantly smaller, 1.6 mm in comparison to 7.38 mm for RtTA1, while for the other tested derivatives, it ranged from 7.5 to 8.25 mm (Fig. 7). This shows that mainly pRleTA1d-located genes are essential for RtTA1 motility.

### The impact of RtTA1 plasmids on the metabolic profile of the cells

To assess the metabolic differences between the RtTA1 wild type and its derivatives with changed plasmid content, Biolog GN2 microplates containing 96 carbon and nitrogen sources were used (Supplementary Table S1). To simplify the analysis, the substrates in GN2 microplates were divided into sugars (polysaccharides, oligosaccharides, monosaccharides, comprising 34.4 % of the tested compounds), modified sugar acids, carboxylic acids (29.2 %), amino acids (21.9 %), nitrogen bases and other compounds (15.5 %) (Table 2). In general, the loss or deletion of any of the plasmids resulted in changes in the metabolic capabilities (Table 2). In the group of sugar substrates, the lack of pRleTA1d in RtTA101 and RtTA105 correlated with deficiency in the use of i-erythritol (sugar alcohol) as a metabolic substrate. RtTA101 and RtTA105 were also not able to use D-psicose, a carbon-3 epimer of D-fructose (Supplementary Table S1). L-rhamnose (6-deoxyhexose), xylitol and D-sorbitol (sugar alcohols) were not used by the RtTA102 strain cured of pRleTA1b. The inability to use gentiobiose, a disaccharide composed of two units of D-glucose, was observed for all RtTA1 derivatives (Supplementary Table S1).

**Table 2:** Metabolic profiles of RtTA1 and its derivatives cured or deleted in plasmids, assayed on Biolog GN2 microplates. The metabolic substrates were divided into four groups. The number of substrates in each group and the number of substrates utilised by strains is shown

	Total substrates	Sugars	Acids	Amino acids	Others
No. of substrates in group	95	35	28	23	9
Strain/genotype	No. of substrates utilised				
RtTA1/wild type	35	21	8	6	0
RtTA101/pd^−^	27	19	6	2	0
RtTA105/pd^−^	28	19	6	3	0
RtTA102/pb^−^	25	20	5	0	0
RtTA104/pa∆	29	18	7	4	0
RtTA106/pa∆	29	20	6	3	0
RtTA109/pa∆	31	21	6	4	0

pd^−^, pb^−^: strains lacking plasmid; pa∆: partial deletion of plasmid pSym

Noticeable changes in the metabolic profiles were observed in the group of tested carboxylic acids. Firstly, a correlation between pRleTA1d elimination and deficiency in using *cis*-aconitic acid was found. Secondly, pRleTA1b elimination or pRleTA1a deletion were correlated with the inability to use γ-hydroxybutyric acid. The most spectacular differences in the number of substrates metabolised by the studied strains were observed in the amino acid group, especially in the case of the pRleTA1b-cured derivative, which was defective in using L-aspartic acid, L-glutamic acid, L-histidine, L-ornithine and L-proline. The pRleTA1d-cured strain did not use L-histidine and L-aspartic acid as substrates. All RtTA1 derivatives under study lost the ability to use L-ornithine and L-proline (Supplementary Table S1).

## Discussion

The extrachromosomal replicons of *R. leguminosarum* bv. *trifolii* TA1 constitute approximately 35.5 % of the total genome. It can be assumed that such a huge amount of accessory genetic information must significantly contribute to the cell phenotype and metabolism. In this work, we succeeded in eliminating individual plasmids pRleTA1b and pRleTA1d and obtained derivatives with partial deletions in pSym. Only pRleTA1c elimination or deletion was unsuccessful. The failed attempts to eliminate the pRleTA1c and pRleTA1a replicons entirely suggest that they are essential for RtTA1 viability under the conditions studied. This was an unexpected result, as the other RtTA1 replicons, i.e. pRleTA1b and pRleTA1d, were previously described as chromid-like, based on in silico analyses and their membership to the same incompatibility group with well-defined chromid replicons of *R. leguminosarum* bv. *viciae* and *R. etli* (Harrison et al. 2010; Mazur et al. 2011a). In turn, on the basis of partial sequence analyses, the pRleTA1c and pRleTA1a replicons were classified as plasmids—less adapted to the host genome and presumably unessential for strain viability (Mazur et al. 2011a).

The genome sequence of RtTA1 has been released only as draft assemblies, precluding precise localisation of important or essential genes in RtTA1 plasmids. Moreover, only a few RtTA1 extrachromosomal markers have been sequenced (Król et al. 2008; Mazur et al. 2011b), allowing only speculation about the importance of the pRleTA1c replicon for cell viability. In this replicon, besides specific *repABC* replication/partition genes, *lps*β2 encoding a dTDP-glucose 4,6-dehydrogenase involved in the biosynthesis of O-antigen, *otsB* encoding a phosphate-trehalose, *tauA* encoding a taurine uptake protein and *orf14* encoding a flavin monooxygenase/reductase protein have been mapped (Król et al. 2007; Mazur et al. 2011b). The homologues of *lps*β2 have been previously described as plasmid located (*lps*β1–*lps*β2 region in pRet42b) and involved in LPS synthesis in *R. etli* (García-de los Santos and Brom 1997) and *R. leguminosarum* bv. *viciae* (Carlson et al. 1995; Young et al. 2006). LPS is the major structural component of the outer membrane of Gram-negative bacteria and the absence of the *lps* genes affects substantially bacterial growth. Thus, the *lps*β2 gene encoding a hypothetical protein involved in the synthesis of 6-deoxy or dideoxy sugars, linking the O-antigen to the core oligosaccharide, could be a reasonable candidate for an essential gene in pRleTA1c, supporting its essentiality for bacterial growth. However, as demonstrated by Brom et al. (2000), pRet42b carrying *lps*β1–*lps*β2 can be entirely eliminated from *R. etli*. To gain further insight into a presence of putative essential genes in pRleTA1c, a 255,620-bp-long contig of this plasmid (available in the GenBank draft genome database, accession no. AKIA01000012), comprising less than half of the plasmid size, was used as a query against the Database of Essential Genes (DEG) (Luo et al. 2013). Among the identified ORFs, those with similarity to proteins constituting hypothetical ABC-type transporters predominated. They may be presumably involved in the transport of peptides (both oligo- and dipeptide), amino acids, maltose/mannitol, iron, spermidine/putrescine and sulphates. Moreover, putative ORFs with similarity to biotin carboxylase subunit and related to glycerol metabolism were found.

In the case of symbiotic plasmid pRleTA1a, which can be lost in other *R. leguminosarum* strains, only partially deleted derivatives were obtained for RtTA1. The result again suggested the presence of essential genes on this plasmid. A similar approach was employed in the search for pRleTA1a putative essential genes. In a 136,237-bp contig of pSym (GenBank accession no. AKIA01000031), a large number of ORFs with sequence similarity to the putative transporters was identified (involved in the transport of calcium, zinc/cadmium/mercury/lead, copper, molybdate, glutamate/aspartate). Furthermore, hypothetical osmolarity response regulator and two-component sensory transduction proteins were detected. In the analysed contig, the *fusA* gene encoding hypothetical elongation factor G (proteins of this family can be involved in the elongation and ribosome recycling during protein synthesis) and the *ftsE* gene encoding a putative cell division ATP-binding protein/septation component were found. Genes related to septum formation were also identified in pRet42e, which cannot be entirely eliminated from *R. etli* (Landeta et al. 2011). Similarly to pRet42e, genes involved in thiamine biosynthesis are located in pRleTA1a (Mazur et al. 2011b). Concluding, in silico analyses of pRleTA1c and pRleTA1a replicons’ partial sequences did not unambiguously prove the essential genes content. However, in the context of the discussion presented by Petersen et al. (2013), pRleTA1c and pRleTA1a could be defined as common plasmids that acquired some chromosomal sequences, e.g. via intragenomic recombination, while pRleTA1b and pRleTA1d are chromid-like (Mazur et al. 2011a) “sensu lato” that are curable and dispensable for survival under laboratory conditions. This implies that no chromids “sensu stricto” (Petersen et al. 2013), which are non-curable and essential for growth under all conditions, were identified in the RtTA1 genome. The completion of several rhizobial genome projects provided numerous pieces of evidence of such intragenomic recombination events, which lead to the location of essential genes on plasmids. The pSymB megaplasmid of *S. meliloti* 1021 contains genes encoding tRNA^arg^ and *engA* (GTPase, likely to be involved in ribosome biogenesis) (diCenzo et al. 2013). The homologous genes of closely related *S. fredii* NGR234 are located on the chromosome, which strongly suggests that they could be translocated from the chromosome to the progenitor of pSymB in an ancestor common to both bacterial species. Other examples of core genes located on plasmids are *panCB* genes responsible for pantothenate biosynthesis in pRet42f, the largest replicon of *R. etli* CFN42 (a second copy of *panB* is also located in pRet42e) (Villaseñor et al. 2011). Pantothenate is considered relevant for central metabolism, and the plasmid location of *pan* genes may be explained by their intragenomic transfer from the chromosome, because *panCB* orthologues are chromosomally located in *R. leguminosarum* bv. *viciae* and *trifolii* and other *Rhizobiales* (Villaseñor et al. 2011).

We demonstrated that both pRleTA1a (pSym) and pRleTA1d (non-symbiotic plasmid) are essential for RtTA1 symbiosis with clover. The pRleTA1d-cured strains induced an even higher number of nodules on clover than the wild type, but did not fix nitrogen. This result not only demonstrated the indispensability of pRleTA1d for successful symbiosis but also pointed out the interactions between the plasmids within the RtTA1 cell, which contribute to important bacterial capabilities. In the pioneer studies with *R. etli* derivatives cured of individual plasmids, Brom et al. (1992) showed that, in addition to pSym (pRet42d), a second, non-symbiotic plasmid (pRet42b) was also indispensable for nodule formation due to the presence of the sequences required for LPS biosynthesis. The homologous *lps*β region in RtTA1 is located in the pRleTA1c non-curable plasmid, whose role in symbiosis remains unknown, rather than in pRleTA1d. However, the presence of some other symbiotically important sequences cannot be precluded in the case of such a large replicon as pRleTA1d. There are several other proofs for the impact of non-symbiotic plasmids on rhizobial interaction with legume plants. In *R. leguminosarum* bv. *viciae* VF39, besides pSym, two other plasmids carry genes necessary for symbiosis, including *lps* genes (Hynes and McGregor 1990). In *R. leguminosarum* bv. *viciae* 3841, the expression of several plasmid genes was induced in the pea rhizosphere; those genes were specifically located on conjugative, non-symbiotic pRL8JI and represented about 15 % of the plasmid genes (Ramachandran et al. 2011).

Elimination of the plasmid from *R. leguminosarum* bv. *trifolii* may affect both saprophytic lifestyle as well as growth in the clover rhizosphere in a natural bacterial population (Moënne-Loccoz and Weaver 1995a, b). This may be a result of the plasmid location of some catabolic genes, such as those involved in the utilisation of rhamnose (Oresnik et al. 1998), erythritol (Yost et al. 2006; Geddes et al. 2010) and glycerol (Ding et al. 2012). Ding et al. (2012) showed that mutants in glycerol transport and catabolism genes located in pRleVF39c of *R. leguminosarum* bv. *viciae* VF39 were not only unable to use glycerol but were also deficient in competitiveness for the nodulation of peas (Ding et al. 2012). In our studies, the metabolic consequences of RtTA1 curing of the largest pRleTA1d were considerable: it not only impaired the ability for nitrogen fixation but also affected the growth kinetics in the complete media. Similarly, curing RtTA1 of the pRleTA1b resulted in deficiency in using several amino acids as metabolic substrates and affected strain growth in the complete media. On the contrary, the growth of RtTA1 Nod^−^ derivatives that lost a substantial part of pSym was comparable to the wild type in complete and minimal media. Similarly, mutations in common *nod* genes did not affect the growth of *S. meliloti* (Fujishige et al. 2008). Furthermore, curing of the pRme2011 symbiotic plasmid of *S. meliloti* (Oresnik et al. 2000) or sequential deletions covering the entire pSymA of *S. meliloti* 1021 (Yurgel et al. 2013) did not affect the free-living growth of bacteria; however, it entailed some non-essential defects in cell catabolism. Consequently, we have demonstrated in the Biolog GN2 test that the metabolic capabilities of the RtTA106, RtTA107 and RtTA109 strains deleted in pSym differed from those of the wild type: the strains did not metabolise amino acids (L-proline, L-ornithine) and acids (succinic acid methyl ester, γ-hydroxybutyric acid). The ability to utilise amino and organic acids is an important attribute of *R. leguminosarum* competitiveness in the rhizosphere. We found in previous studies that the most competitive rhizobial strains were those capable of using a broad range of amino and organic acids (Wielbo et al. 2007, 2010). Mutants unable to use L-homoserine (which is the component of pea root exudate) as the sole carbon source were affected in the competitive nodulation of pea and lentil (Vanderlinde et al. 2014). Furthermore, strong up-regulation of some plasmid-encoded genes of *R. leguminosarum* bv. *viciae* 384 in the pea rhizosphere was demonstrated by a microarray-based study (Ramachandran et al. 2011).

The RtTA1 derivatives deleted in pSym exhibited substantially reduced biofilm formation abilities (on average 66 % of the wild type). The development of biofilm requires the expression of numerous genes implicated in various cell processes, like signalling, stress responses, motility and the synthesis of structures responsible for cell attachment. Our result is consistent with the observation made by Fujishige et al. (2008): in *S. meliloti*, products of common *nodABCD* genes, responsible for core NF synthesis, were also required for rhizobial cell-to-surface adhesion. The core NF structure is similar to chitosan, which promotes cell adhesion by making the microbial surface more hydrophobic (Fujishige et al. 2008). The smallest amount of biofilm (57 %) was formed by a strain cured of pRlTA1b, indicating that genes located on this plasmid play a role in biofilm formation, together with the ones from pSym. On the contrary, the largest plasmid-cured derivatives produced 80–88 % biofilm compared to the RtTA1 wild type, demonstrating an insignificant role of this plasmid in biofilm formation. The biofilm analyses once again revealed the interrelation among the plasmids residing in one cell.

Autoaggregation is yet another common phenotype of bacteria that have a strong tendency toward adhesive interactions among cells. This is manifested by clumping in liquid cultures (Sorroche et al. 2012) and several surface components, such as LPS, outer membrane or pili, have been described as adhesion conducive (Sorroche et al. 2010). Autoaggregation could reflect a survival strategy of bacteria under stress environmental conditions. From among the RtTA1 derivatives, RtTA102 cured of the pRleTA1b plasmid displayed the strongest autoaggregation tendency in relation to the wild type. Taking into account the fact that RtTA102 is severely defective in overall metabolism, the suggestion about the influence of stress conditions on aggregation seems to be supported.

A majority of the hitherto identified genes required for EPS synthesis have been mapped on chromosomes of *R. leguminosarum* and *R. etli* (González et al. 2006; Young et al. 2006; Mazur et al. 2011b), but some putative EPS/LPS synthesis genes were also found to be scattered among plasmids (Król et al. 2007). Loss of the pRleTA1b plasmid with a Pss-III region did not influence significantly the level of EPS synthesis in RtTA102, demonstrating an irrelevant role of this plasmid in EPS biosynthesis. Nevertheless, the generally increased production of EPS by all the RtTA1 derivatives studied suggested a complex regulatory network of EPS synthesis, presumably comprising genes located in different plasmids.

In several studies, a relation between the production of bacterial polysaccharides and motility has been shown. Motility is required for the chemotactic movement of bacteria towards the compounds released by its host, while exopolysaccharides are needed for bacterial attachment to the root or for invasion of the infection thread (Bahlawane et al. 2008; Morris and González 2009). The swarming ability of *S. meliloti* Rm2011 was shown to depend on a functional ExpR/Sin quorum-sensing system and the production of both flagella and EPS (Hoang et al. 2008). In *R. leguminosarum* bv. *viciae*, six flagellin genes (*flaA*/*B*/*C*/*D*/*H*/*G*) were found on the chromosome: *flaA*/*B*/*C*/*D* are located within the major chemotaxis and the motility cluster, while *flaE* was mapped on plasmid pRle11JI (Tambalo et al. 2010). In this work, the pRleTA1d-cured derivatives are non-motile, indicating a possibility of the regulation of putative flagellin gene expression by the plasmid-located loci. However, further experimentation is needed to confirm the correlation between motility and the plasmid gene(s).

Overall, our approach, whose aim was to eliminate individual plasmids from *R. leguminosarum* bv. *trifolii*, revealed their functional significance and indispensability in some metabolic pathways, symbiotic interaction with host plant, biofilm formation and potential survival in the soil. Equally important, the results suggested a broad relationship among the plasmids in shaping the symbiotic capabilities and cell phenotype.

## Electronic supplementary material

Below is the link to the electronic supplementary material.

Supplementary Table S1 The raw data for the phenotypic profiling of RtTA1 and its derivatives cured or deleted in plasmids, using Biolog GN2 microplates. The data were binary coded for statistical analyses: conversion of tetrazolium violet to a purple-coloured compound was considered as a positive phenotype (coded “1”, marked in red); in turn, when the wells remained colourless, the phenotype was considered negative (coded “0”). (XLSX 13 kb)
